# SET Domain Genes in Neurodevelopmental Disorders

**DOI:** 10.3390/genes17101313

**Published:** 2026-10-21

**Authors:** Aki Ohguri, Tadashi Nakagawa, Makiko Nakagawa, Toru Hosoi

**Affiliations:** 1 Department of Clinical Pharmacology, Faculty of Pharmaceutical Sciences, Sanyo-Onoda City University, Sanyo-Onoda 756-0884, Japan; p123016@ed.socu.ac.jp (A.O.); hosoi@rs.socu.ac.jp (T.H.); 2 Institute of Gene Research, Yamaguchi University Science Research Center, Ube 755-8505, Japan; mnakagaw@yamaguchi-u.ac.jp; 3 Advanced Technology Institute, Life Science Division, Yamaguchi University, Ube 755-8611, Japan

**Keywords:** SET domain, neurodevelopmental disorders, histone methylation, epigenetic regulation, chromatin regulation, intellectual disability

## Abstract

Neurodevelopmental disorders (NDDs) comprise a genetically and clinically heterogeneous group of conditions, with disruption of chromatin regulation and developmental gene-expression programs representing an important molecular mechanism underlying their pathogenesis. SET domain-containing (SETD) proteins constitute a diverse family of chromatin regulators that primarily mediate histone methylation, although some lack demonstrated intrinsic catalytic activity or modify non-histone substrates. Pathogenic variants in multiple SETD genes, including *SETD1A*, *KMT2D*, *SETD2*, *NSD1*, and *EZH2*, cause NDDs characterized by developmental delay, intellectual disability, speech and language impairment, autism-related features, epilepsy, growth abnormalities, and congenital anomalies. Representative examples include SETD1A-related neurodevelopmental disorder (OMIM #619056), KMT2D-related Kabuki syndrome type 1 (OMIM #147920), SETD2-related Luscan–Lumish syndrome (OMIM #616831), NSD1-related Sotos syndrome (OMIM #117550), and EZH2-related Weaver syndrome (OMIM #277590). In this review, we summarize the classification, domain architecture, and molecular functions of human SETD proteins and integrate clinical findings with mechanistic insights obtained from genetically modified mouse models. These studies reveal substantial cross-species convergence in cognitive, behavioral, synaptic, and developmental phenotypes while also demonstrating that individual SETD proteins regulate distinct molecular processes. Importantly, phenotypic similarity does not necessarily indicate a common molecular mechanism, and variant-specific effects, including loss-of-function, gain-of-function, dominant-negative, and substrate-selective alterations, must be considered when interpreting experimental models. Several studies further demonstrate partial reversibility of molecular and behavioral abnormalities through restoration of SETD protein function, inhibition of opposing chromatin-modifying enzymes such as LSD1, or correction of downstream signaling pathways. These findings suggest that therapeutic opportunities may extend beyond transcriptional regulation to downstream processes including translation, synaptic function, and cellular signaling. Integrating human genetics with animal and human cellular models will therefore be essential for defining shared and gene-specific mechanisms and identifying therapeutically actionable pathways in SETD-related NDDs.

## 1. Introduction

Neurodevelopmental disorders (NDDs) comprise a heterogeneous group of conditions characterized by disturbances in cognitive, behavioral, social, language, or motor development [1,2]. Large-scale sequencing studies have identified numerous risk genes involved in chromatin organization and transcriptional regulation, supporting the concept that disruption of developmental gene-expression programs represents a major route to NDD pathogenesis [3,4,5,6,7]. Among these regulators, genes encoding SET domain-containing (SETD) proteins have emerged as an important group associated with developmental abnormalities.

**Figure 1:**
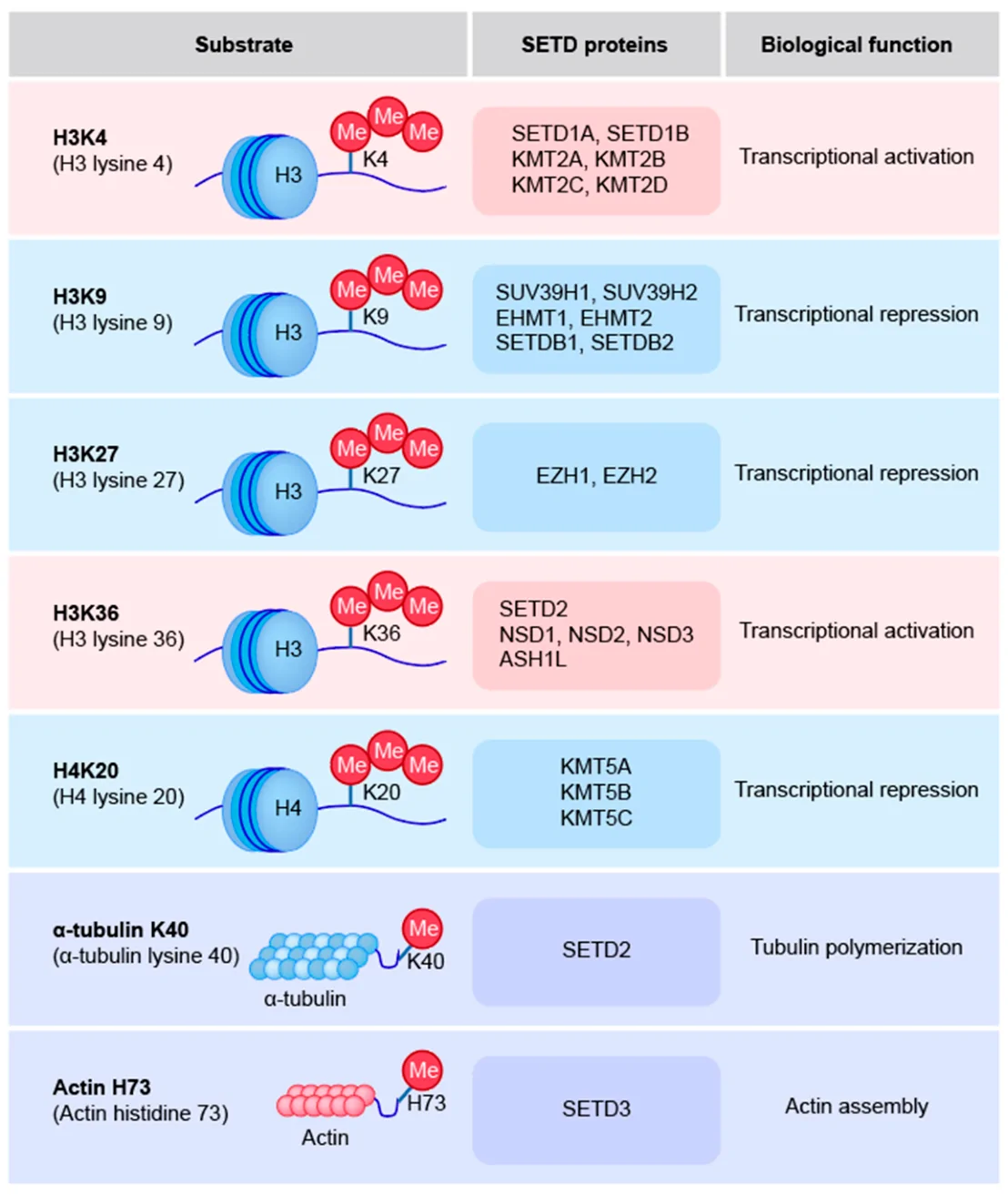
Substrate specificity and context-dependent regulatory functions of human SETD proteins discussed in this review. SETD1/KMT2 family members catalyze histone H3 lysine 4 (H3K4) methylation. SUV39/EHMT/SETDB family proteins catalyze histone H3 lysine 9 (H3K9) methylation. EZH1 and EZH2 catalyze histone H3 lysine 27 (H3K27) methylation. SETD2/NSD/ASH1L family proteins catalyze histone H3 lysine 36 (H3K36) methylation. KMT5 family members catalyze histone H4 lysine 20 (H4K20) methylation. In addition to histone substrates, SETD2 and SETD3 catalyze the methylation of α-tubulin at lysine 40 (K40) and actin at histidine 73 (H73), respectively. Red backgrounds indicate histone modifications associated with transcriptional activation, and blue backgrounds indicate those associated with transcriptional repression in general. Of note, the regulatory functions of these modifications depend on their methylation states, genomic distributions, and chromatin contexts and are not restricted to transcriptional activation or repression. Purple backgrounds indicate methylation events involved in cytoskeletal regulation. Me, methyl group.

The SET domain derives its name from the Drosophila proteins Suppressor of variegation 3–9, Enhancer of zeste, and Trithorax, which catalyze the methylation of specific lysine residues on histones [8]. Histone lysine methylation contributes to the regulation of chromatin organization and transcription through mechanisms that depend on the modified residue, degree of methylation, genomic localization, and cellular context. Although H3K4 and H3K36 methylation are generally associated with transcriptionally permissive chromatin, whereas H3K9, H3K27, and H4K20 methylation are frequently linked to transcriptional repression, these associations do not imply strictly activating or repressive functions [9,10]. For example, H3K4me3 is predominantly enriched at active promoters, whereas H3K4me1 is commonly associated with enhancers, including both active and poised regulatory elements [11]. Similarly, H3K36me3 is enriched within transcribed gene bodies, where it contributes to transcriptional fidelity by suppressing cryptic intragenic transcription [12]. Furthermore, H3K36me2/3 can antagonize PRC2-mediated H3K27 methylation [13,14], illustrating the functional interplay between distinct histone modifications. Thus, the biological consequences of histone methylation cannot be inferred solely from the identity of the modified residue but must be interpreted in the context of its genomic distribution and associated regulatory machinery (Figure 1).

A subset of SETD proteins lack demonstrated intrinsic catalytic activity, whereas others methylate non-histone substrates or target amino acid residues other than lysine. The SET domain is a conserved protein domain comprising approximately 130–140 amino acid residues and is annotated in established protein-domain databases, including InterPro (IPR001214) and PROSITE (SET domain profile, PS50280). Although SET domains share a conserved three-dimensional fold that is fundamentally distinct from that of conventional methyltransferases [15,16], their primary sequences exhibit considerable divergence among different protein families. In catalytically active SETD proteins, the SET domain mediates methyl-group transfer, typically using S-adenosylmethionine (SAM) as a methyl-group donor. Previous sequence and structural analyses have identified conserved motifs and residues involved in SAM binding, substrate recognition, and methyl-group transfer [17]. Notably, comparative analyses of SET domains from representative methyltransferases, including KMT2A, KMT2C, NSD1, and ASH1L, have demonstrated that KMT2E and SETD5 exhibit substantial divergence at residues implicated in catalytic activity and cofactor binding [18]. These observations are consistent with the absence of detectable intrinsic methyltransferase activity for KMT2E under the experimental conditions examined and the lack of firmly established intrinsic catalytic activity for SETD5. Thus, despite conservation of the overall SET-domain architecture, variations in amino acid sequences and residues associated with catalysis contribute to the functional diversity of SETD proteins. Importantly, the presence of an annotated SET domain does not necessarily indicate intrinsic methyltransferase activity or equivalent substrate specificity.

**Figure 2:**
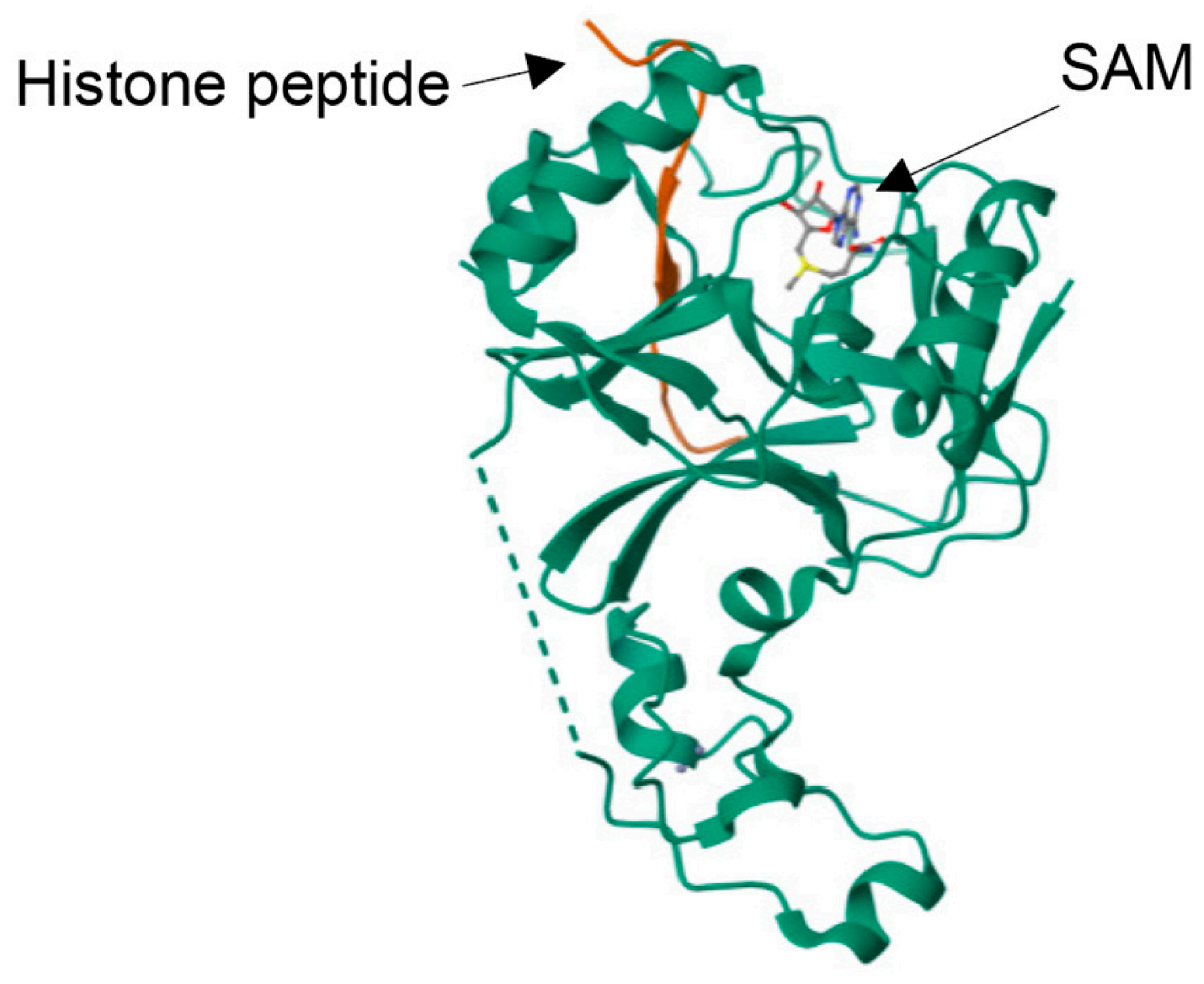
Three-dimensional structure of the human SETD2 SET domain. Ribbon representation of the experimentally determined crystal structure of human SETD2 in complex with an H3K36M histone peptide and S-adenosylmethionine (SAM) (PDB ID: 5V21). The structure was determined by X-ray crystallography at a resolution of 2.42 Å. Bound histone peptide and SAM are indicated.

The three-dimensional organization of the SET domain is illustrated by the experimentally determined crystal structure of human SETD2 in complex with an H3K36M histone peptide and SAM (PDB ID: 5V21; Figure 2) [19]. This structure, resolved at 2.42 Å by X-ray crystallography, provides insights into the structural organization of the SET domain and the molecular basis of cofactor binding and histone substrate recognition.

Experimental mouse models carrying mutations in SETD genes provide an essential complement to human genetic studies by enabling systematic investigation of the molecular, cellular, and developmental consequences of altered SETD protein function. Such models allow candidate disease mechanisms to be examined under controlled genetic conditions and facilitate determination of the developmental stages, tissues, and cell types in which individual genes exert their critical functions [20,21].

In this review, we summarize the classification, domain architecture, and evolutionary relationships of human SETD proteins and discuss the neurodevelopmental phenotypes associated with SETD gene mutations in humans and mice. This review focuses on SETD protein families that include at least one member with an established association with NDDs. Accordingly, families and individual proteins without established NDD associations, such as the PRDM family, SETD6, SETD7, and SETMAR, are not discussed. Nevertheless, selected proteins without established NDD associations are included to illustrate the structural and functional diversity within NDD-associated SETD protein families and to facilitate comparisons among family members. By integrating molecular, experimental, and clinical evidence, this review aims to provide a framework for understanding the biological basis of SETD protein-related NDDs and to identify priorities for future mechanistic studies and therapeutic development.

## 2. SETD1/KMT2 and Its Related Families

**Figure 3:**
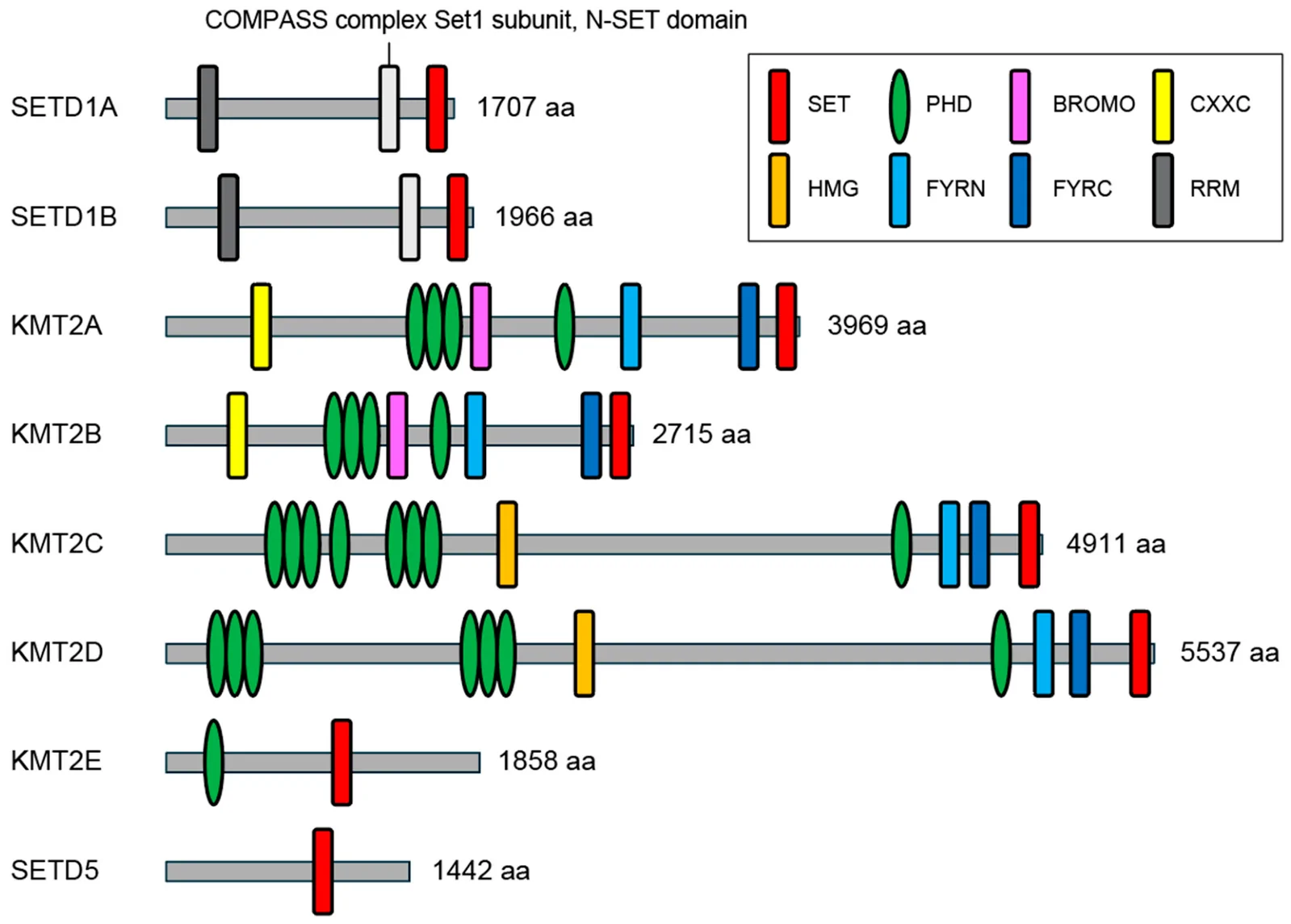
Domain structures of SETD1/KMT2 family histone methyltransferases. SET domains are indicated by red rectangles, PHD domains by green ellipses, bromodomains (BROMO) by pink rectangles, CXXC domains by yellow rectangles, HMG domains by orange rectangles, FYRN domains by light blue rectangles, and FYRC domains by blue rectangles. RNA recognition motif (RRM) domains and COMPASS complex Set1 subunit N-terminal (N-SET) domains are indicated by gray and light gray rectangles, respectively.

The SETD1/KMT2 family comprises six principal human H3K4 methyltransferases: SETD1A, SETD1B, KMT2A (also known as MLL1), KMT2B (also known as MLL2), KMT2C (also known as MLL3), and KMT2D (also known as MLL4) (Figure 1 and Figure 3). These proteins form three evolutionarily related pairs corresponding to the Drosophila Set1 (SETD1A and SETD1B), Trithorax (KMT2A and KMT2B), and Trithorax-related (KMT2C and KMT2D) branches [22,23,24]. Their catalytic SET domains are located near the C terminus and function within multiprotein COMPASS or COMPASS-like complexes containing the conserved core components WDR5, RBBP5, ASH2L, and DPY30, or their evolutionary counterparts. The organization and H3K4 methyltransferase function of these complexes are evolutionarily conserved from yeast to mammals, although their catalytic activities and methylation-state preferences have diversified. In *Saccharomyces cerevisiae*, Set1 is the principal H3K4 methyltransferase and catalyzes mono-, di-, and trimethylation within the COMPASS complex [25,26]. In *Drosophila melanogaster*, three related methyltransferases, dSet1, Trithorax (Trx), and Trithorax-related (Trr), function in distinct COMPASS-like complexes. Among these, dSet1 is primarily responsible for global H3K4 di- and trimethylation [27,28], whereas Trr preferentially contributes to enhancer-associated H3K4 monomethylation [29]. Mammals possess six corresponding methyltransferases organized into three major subfamilies: SETD1A/SETD1B, KMT2A/KMT2B, and KMT2C/KMT2D. Consistent with their evolutionary relationships, SETD1A/SETD1B complexes contribute substantially to H3K4 trimethylation, particularly at gene promoters [30,31,32], whereas KMT2C/KMT2D complexes are major regulators of enhancer-associated H3K4 monomethylation [32,33,34]. KMT2A/KMT2B complexes exhibit more context-dependent activities, contributing to H3K4 methylation at selected genomic loci [32,35,36]. Furthermore, biochemical reconstitution studies of human SETD1/KMT2 complexes have demonstrated differences in intrinsic catalytic activity, dependence on conserved regulatory subunits, and the relative production of H3K4me1, H3K4me2, and H3K4me3 [37]. Thus, although H3K4 is a conserved substrate across species, the relative contributions of individual COMPASS-family complexes to different methylation states and genomic regions have diversified during evolution. These differences may contribute to the distinct developmental functions and neurodevelopmental phenotypes associated with mutations in individual SETD1/KMT2 family members.

Although all six members of the mammalian SETD1/KMT2 H3K4 methyltransferase family have been implicated in human developmental disease, pathogenic variants in these genes give rise to overlapping yet distinct phenotypic spectra (Table 1). *SETD1A* haploinsufficiency is associated with developmental delay or intellectual disability, prominent speech and language difficulties, behavioral abnormalities, and variable epilepsy [38] (OMIM# 619056). *Setd1a*-deficient mice exhibit working-memory impairment accompanied by reduced axonal branching and abnormalities in cortical synaptic dynamics [39]. Independent studies have also identified impaired excitatory synaptic function and schizophrenia-related behavioral abnormalities [40]. Genomic analyses demonstrated that SETD1A occupies both promoters and enhancers, with substantial overlap between SETD1A and the neuronal transcription factor MEF2 at enhancer regions [39]. SETD1A target genes are highly expressed in cortical pyramidal neurons and exhibit a complex combination of increased and decreased expression following *Setd1a* deficiency, consistent with distinct regulatory functions of SETD1A at promoters and MEF2-associated enhancers. Evolutionarily conserved SETD1A targets are also enriched for genes carrying neuropsychiatric genetic risk, providing a molecular connection between SETD1A-dependent transcription and disease susceptibility. Importantly, restoring Setd1a expression in adulthood ameliorates working-memory deficits, while inhibition of the counteracting lysine demethylase LSD1 rescues both behavioral and morphological abnormalities [39].

SETD1B-related NDD is characterized by developmental and language delay, ID, autism or other behavioral difficulties, and heterogeneous epilepsy phenotypes [41] (OMIM#619000) (Table 1). Developmental impairment can precede seizure onset, indicating that the cognitive phenotype cannot be attributed solely to epileptic activity. Conditional deletion of *Setd1b* in excitatory neurons of the postnatal forebrain causes severe impairments in hippocampus-dependent learning and spatial memory [69]. Neuron-specific ChIP-seq and RNA-seq demonstrated that *Setd1b* loss preferentially reduces H3K4me3 at promoters characterized by broad H3K4me3 domains and decreases expression of highly expressed neuron-enriched genes involved in synaptic plasticity, learning, and memory [69]. Comparison with *Kmt2a*- and *Kmt2b*-deficient mice (discussed below) further showed that SETD1B exerts a particularly strong influence on this class of neuronal genes. These observations indicate that SETD1A/SETD1B are not merely required to establish developmental transcriptional programs but continuously maintain a specialized epigenetic state necessary for mature neuronal function.

Heterozygous pathogenic variants in *KMT2A* cause Wiedemann–Steiner syndrome (OMIM#605130), characterized by developmental delay, variable intellectual disability, speech impairment, and hypotonia, often accompanied by hypertrichosis, short stature, feeding difficulties, and distinctive facial features [42,43] (Table 1). *KMT2B*-related disease (OMIM#617284, 619934) encompasses both childhood-onset progressive dystonia and a broader neurodevelopmental phenotype. Dystonia frequently begins in the lower limbs and subsequently generalizes, with prominent cranial and laryngeal involvement. Developmental delay, intellectual disability, microcephaly, and growth restriction may coexist, whereas some individuals present with developmental abnormalities without dystonia [44] (Table 1). Conditional depletion of either *Kmt2a* or *Kmt2b* in forebrain neurons impairs memory, but comparative ChIP-seq and RNA-seq analyses revealed that the two enzymes predominantly regulate distinct genomic regions and molecular pathways associated with neuronal plasticity [70,71]. Thus, apparently similar cognitive phenotypes can arise from disruption of different transcriptional networks. More recently, a genetically rescuable heterozygous *Kmt2a* model reproduced growth and craniofacial abnormalities together with hippocampal memory impairment [72]. Restoration of KMT2A during embryonic development rescued neurological phenotypes, supporting an important developmental contribution to disease pathogenesis [72]. The relationship between human and mouse phenotypes also illustrates an important limitation of cross-species modeling. Although developmental and cognitive abnormalities observed in *Kmt2a*- and *Kmt2b*-deficient mice correspond broadly to the neurodevelopmental manifestations of the respective human disorders, these models do not necessarily reproduce their complete clinical spectra. In particular, the forebrain *Kmt2b* models described above predominantly exhibit cognitive abnormalities rather than the progressive dystonia that represents a major manifestation of KMT2B-related disease. Such discrepancies may reflect species-specific developmental requirements, compensatory mechanisms, or differences between the molecular consequences of experimental loss-of-function alleles and those of individual human pathogenic variants.

Heterozygous loss-of-function variants in *KMT2C* cause a variable disorder involving developmental delay, intellectual disability, language impairment, and behavioral or psychiatric difficulties, with autism and seizures in a subset of individuals [45] (Table 1). Although historically designated Kleefstra syndrome type 2 (OMIM#617768) [73], recent clinical and DNA-methylation analyses support recognition of KMT2C-related NDD as distinct from Kleefstra syndromes [45]. By contrast, heterozygous loss-of-function variants in *KMT2D* are the cause of Kabuki syndrome type 1 (OMIM#147920), in which developmental delay and commonly mild-to-moderate intellectual disability occur alongside characteristic facial features, persistent fingertip pads, skeletal abnormalities, postnatal growth deficiency, and variable cardiac, auditory, and immune abnormalities [46,47,48] (Table 1). Interestingly, selected missense variants in a restricted region of KMT2D produce a multiple-malformation disorder distinct from typical Kabuki syndrome [74], illustrating the importance of variant location and mechanism. Mice carrying a heterozygous mutation in *Kmt2c* exhibit memory deficits and other autism spectrum disorder-related abnormalities [75]. Bulk-brain RNA-seq revealed enrichment of autism spectrum disorder-risk genes among upregulated transcripts, whereas genomic KMT2C-binding sites were preferentially associated with downregulated genes [75], suggesting that both direct and indirect transcriptional consequences contribute to the phenotype. Single-cell RNA-seq of neonatal brains further localized strong autism spectrum disorder-associated transcriptional abnormalities to radial glial cells and immature neurons despite the absence of major changes in overall cellular composition [75]. Similar to *Setd1a* mutant mice [39], treatment of adult *Kmt2c* mutant mice with an LSD1 inhibitor improves social behavior and shifts the majority of altered transcripts toward wild-type expression levels [75]. Heterozygous *Kmt2d* mutant mice exhibit reduced H3K4me3 in the dentate gyrus, impaired adult hippocampal neurogenesis, and deficits in hippocampus-dependent memory [76,77]. Treatment with either a histone deacetylase inhibitor or an LSD1 inhibitor restores aspects of chromatin accessibility, improves neurogenesis, and ameliorates memory impairment [76,77]. Although these studies do not compare the gene-expression changes in Kmt2d mutant mice with those of other KMT2-family mutant mice, they support a mechanistic sequence in which *KMT2D* haploinsufficiency alters chromatin regulation, thereby compromising the functional integration of newly generated hippocampal neurons. These findings also illustrate an important aspect of cross-species convergence. Intellectual disability and learning difficulties associated with SETD1A-, KMT2C-, and KMT2D-related disorders are paralleled by impairments in learning, memory, behavioral flexibility, or social behavior in corresponding mouse models. However, similar behavioral outcomes do not necessarily indicate equivalent underlying mechanisms. Working-memory impairment in Setd1a-deficient mice is associated with abnormalities in cortical axonal branching and synaptic dynamics, whereas cognitive dysfunction in *Kmt2d* mutants is closely associated with impaired adult hippocampal neurogenesis. Moreover, the improvement of behavioral and molecular abnormalities following restoration of SETD1A activity or pharmacological modulation of LSD1 or histone deacetylases suggests that at least some consequences of disrupted chromatin regulation remain functionally modifiable beyond early development. Nevertheless, rescue of selected phenotypes in mice should not be interpreted as evidence that corresponding clinical manifestations are necessarily reversible in affected individuals.

KMT2E (also known as MLL5) requires separate consideration. Although its nomenclature places it within the KMT2 family, KMT2E is not one of the six canonical catalytic SET1/COMPASS methyltransferases. KMT2E contains a PHD finger and a structurally divergent SET domain. Structural and biochemical analyses have identified alterations in regions required for substrate and cofactor accommodation and have failed to detect intrinsic methyltransferase activity under the conditions examined. KMT2E therefore illustrates that the presence of a SET domain—or classification within a methyltransferase-related gene family—is not, by itself, sufficient to establish enzymatic activity [17,18]. Heterozygous loss-of-function variants in the *KMT2E* gene cause O’Donnell–Luria–Rodan syndrome (OMIM#618512), characterized primarily by developmental delay/intellectual disability, speech delay, hypotonia, macrocephaly, and variable autism and epilepsy [49,50] (Table 1). Most pathogenic variants are truncating and are consistent with haploinsufficiency, whereas some missense variants are associated with a more severe phenotype including microcephaly and developmental and epileptic encephalopathy [49,50]. *Kmt2e* heterozygous mice exhibit impaired social behavior and increased anxiety-like behavior, accompanied by altered glucose metabolism and neuronal development in the amygdala [78]. More recent studies further demonstrated that *Kmt2e* haploinsufficiency impairs microglial phagocytosis and synaptic clearance through dysregulation of TREM2-SGK3-GSK3β signaling, resulting in neuronal hyperexcitability and autism spectrum disorder-like behaviors [79]. These findings provide experimental support for the neurodevelopmental consequences of *KMT2E* haploinsufficiency observed in humans.

SETD5 represents another structurally unusual member of the SET domain protein family and is particularly relevant to NDDs. SETD5 is evolutionarily related to KMT2E and contains an N-terminal SET domain together with additional regions implicated in transcriptional regulation and protein–protein interactions [80]. Although SETD5 has been implicated in the regulation of H3K36 methylation [81], its intrinsic catalytic activity remains uncertain [18,82,83]. Instead, biochemical and functional evidence indicates that at least some of its effects on chromatin and transcription are mediated through noncatalytic mechanisms, including interactions with chromatin-regulatory complexes such as HDAC3-containing corepressor machinery [80]. Thus, similar to KMT2E, SETD5 exemplifies how SET domain-containing proteins can exert substantial regulatory effects on chromatin and transcription independently of demonstrable intrinsic methyltransferase activity. Haploinsufficiency of *SETD5* causes a variably expressed NDD involving intellectual disability, speech and motor delay, hypotonia, and behavioral abnormalities, including autistic features in some individuals [51,52] (OMIM#615761) (Table 1). Dysmorphism, feeding difficulties, and congenital anomalies also occur [84,85]. *Setd5* heterozygous mice display cognitive impairment, behavioral inflexibility, altered ultrasonic vocalizations, hyperactivity, and abnormalities in social behavior, although the precise phenotype varies among models [81,82,86,87]. Transcriptomic analysis of the developing *Setd5* mutant cortex identified altered expression of neurodevelopment-related genes in specific neuronal populations, accompanied by reduced neurite outgrowth, decreased synaptic density, impaired network activity, and abnormalities in deep-layer cortical neuron development [86]. Other studies have linked *SETD5* deficiency to altered transcriptional elongation and RNA maturation, resulting in aberrant gene expression and splicing during neural development [81]. An additional mechanism involves ribosome biogenesis: RNA-seq of *Setd5* mutant brains revealed preferential reduction in ribosomal protein genes, while rDNA transcription, global translational activity, cyclin D1 translation, and neural-cell proliferation were impaired by *SETD5* insufficiency [87]. SETD5 was shown in this context to regulate rDNA chromatin through recruitment of an HDAC3-containing complex. Thus, *SETD5* deficiency may disturb neurodevelopment through several interconnected processes, including transcriptional fidelity, ribosome biogenesis, protein synthesis, neural progenitor proliferation, and neuronal network formation. At the phenotypic level, cognitive impairment, behavioral inflexibility, abnormalities in social behavior, and altered ultrasonic vocalizations in *Setd5*-deficient mice parallel selected cognitive, behavioral, and communication-related manifestations of SETD5-associated NDD [52].

Taken together, the SETD1/KMT2-related proteins demonstrate particularly strong cross-species convergence in cognitive function. Human disorders caused by pathogenic variants in SETD1A, SETD1B, KMT2A, KMT2C, KMT2D, KMT2E, and SETD5 commonly involve intellectual or developmental impairment, whereas corresponding mouse models frequently exhibit abnormalities in learning, memory, behavioral flexibility, social behavior, or neuronal communication. Importantly, however, this phenotypic convergence occurs despite substantial mechanistic diversity, ranging from altered enhancer and promoter regulation to impaired adult neurogenesis, ribosome biogenesis, synaptic function, and microglial regulation. Cross-species correspondence is therefore generally strongest at the level of affected neurobiological functions rather than complete clinical syndromes.

## 3. SETD2/NSD/ASH1L Family

**Figure 4:**
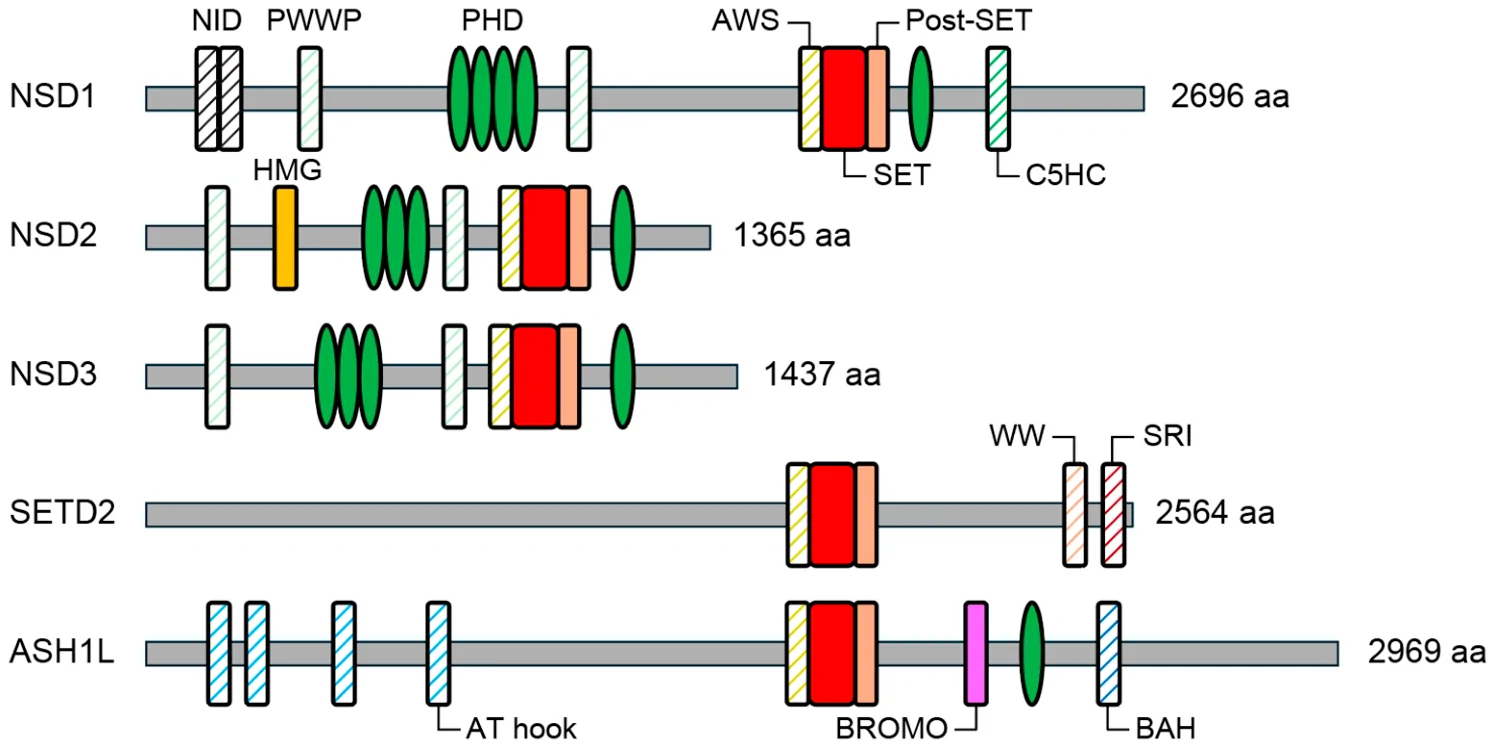
Domain structures of SETD2/NSD/ASH1L family histone methyltransferases. SET domains are indicated by red rectangles, PHD domains by green ellipses, bromodomains (BROMO) by pink rectangles, HMG domains by orange rectangles, nuclear receptor interaction domains (NID) by white rectangles with black diagonal lines, PWWP domains by white rectangles with light green diagonal lines, AWS domains by yellow rectangles, post-SET domains by light red rectangles, C5HC domains by white rectangles with green diagonal lines, WW domains by white rectangles with yellow diagonal lines, SRI domains by white rectangles with red diagonal lines, AT-hook domains by white rectangles with light blue diagonal lines, and BAH domains by white rectangles with blue diagonal lines.

Proteins catalyzing H3K36 methylation include SETD2, NSD1, NSD2, NSD3, and ASH1L (Figure 4). NSD family proteins contain multiple chromatin-interaction modules, including PWWP and PHD domains, surrounding an AWS–SET–post-SET catalytic region. Structural studies of NSD2 and NSD3 bound to nucleosomes have demonstrated that substrate recognition involves rearrangement of nucleosomal DNA and precise positioning of the catalytic domain relative to histone H3, highlighting the importance of nucleosome architecture in regulating their enzymatic activity [88]. ASH1L similarly contains an AWS–SET–post-SET catalytic region, together with a bromodomain, a PHD finger, and a BAH domain. Its catalytic activity is constrained by an autoinhibitory loop that restricts substrate access to the active site. Direct interaction with MRG15 can relieve this autoinhibition by displacing the regulatory loop, thereby enhancing ASH1L-mediated H3K36 methylation [89]. SETD2, by contrast, is distinguished by its close coupling to transcription and its predominant role in catalyzing H3K36 trimethylation. Its conserved Set2–Rpb1 interaction (SRI) domain mediates association with RNA polymerase II, illustrating how accessory domains can couple histone methyltransferase activity to specific cellular processes such as transcriptional elongation [90]. Through its SRI and SET domains, SETD2 also catalyzes the methylation of α-tubulin at lysine 40, thereby contributing to microtubule stabilization [91,92] (Figure 1 and Figure 4).

Pathogenic variants in SETD2 are associated with a spectrum of autosomal dominant neurodevelopmental disorders, including Luscan–Lumish syndrome (LLS) (OMIM#616831), Rabin–Pappas syndrome (RPS) (OMIM#620155), and intellectual developmental disorder, autosomal dominant 70 (MRD70) (OMIM#620157) (Table 1). LLS is primarily associated with heterozygous loss-of-function variants and is characterized by developmental delay, intellectual disability, autism spectrum disorder, macrocephaly, and variable generalized overgrowth [53,54]. In contrast, RPS, most commonly associated with the recurrent missense variant p.Arg1740Trp, manifests as a more severe disorder characterized by profound intellectual disability, microcephaly, growth restriction, and multiple congenital anomalies [55]. Additional missense variants, including p.Glu1718Lys and p.Asp2251Glu, have also been reported in individuals with RPS-like phenotypes [93]. Notably, the p.Arg1740Gln variant is associated with MRD70, which is characterized by intellectual disability and speech impairment without the pronounced growth abnormalities or multiple congenital anomalies typically observed in RPS [55]. Although these disorders share neurodevelopmental impairments, their distinctive clinical presentations and DNA methylation profiles suggest variant-specific effects on SETD2-mediated epigenetic regulation. Together, these findings highlight the complex genotype–phenotype relationships associated with SETD2 dysfunction. Conditional deletion of *Setd2* in the developing dorsal forebrain disrupts cortical arealization and cortico-thalamo-cortical circuit formation, resulting in impaired social interaction, motor learning, and spatial memory [94]. Loss of SETD2-dependent H3K36me3 alters developmental transcription, including reduced expression of clustered protocadherin genes, providing a plausible link between chromatin dysregulation, neuronal recognition and connectivity, and abnormal circuit formation [94]. In contrast, a heterozygous *Setd2* SRI-domain missense model selectively disrupts SETD2-dependent α-tubulin methylation while preserving histone H3K36 methylation. These animals exhibit anxiety-like behavior and primary neurons show reduced axonal length and abnormal dendritic arborization, yet RNA-seq and ChIP-seq detect no substantial accompanying changes in gene expression or chromatin methylation [95]. This model is particularly informative because it demonstrates that SETD2-associated neurobehavioral phenotypes can arise through a non-histone cytoskeletal mechanism independently of detectable transcriptional dysregulation. The marked genotype-dependent clinical heterogeneity of SETD2-related disorders is particularly important when interpreting these mouse models.

Heterozygous *NSD1* variants or deletions cause Sotos syndrome, in which learning difficulties or intellectual disability accompany macrocephaly, overgrowth, advanced bone age, and characteristic facial features [56] (OMIM# 117550) (Table 1). *Nsd1* heterozygous mice exhibit reduced social-novelty preference and decreased pup ultrasonic vocalizations but do not reproduce the generalized somatic or brain overgrowth characteristic of human Sotos syndrome [96]. More mechanistically informative results have emerged from conditional *Nsd1* deletion in the neocortex. Loss of *Nsd1* shifts the boundaries of cortical functional areas, disrupts cortico-thalamo-cortical connectivity, and produces deficits in spatial memory, motor learning, and motor coordination [97]. At the molecular level, *Nsd1* loss causes widespread depletion of H3K36me2 and redistribution of DNA methylation. Superficial-layer pyramidal neurons progressively misexpress deep-layer neuronal markers and fail to achieve normal morphological and electrophysiological maturation [97]. Expression of neocortical layer-specifying genes is reduced, whereas normally silenced non-neural genes become ectopically expressed [97]. NSD1-dependent H3K36me2 therefore appears to stabilize region- and layer-specific transcriptional identities, providing a direct connection between chromatin organization, neuronal identity, circuit assembly, and behavior.

*NSD2* deficiency causes Rauch–Steindl syndrome, typically involving prenatal and postnatal growth restriction, speech and motor delay, and variable, often relatively mild, intellectual impairment [57,58] (OMIM #619695) (Table 1). Although NSD2 lies within the chromosome 4p16.3 region deleted in Wolf–Hirschhorn syndrome, characterized by typical facial appearance, intellectual disability, hypotonia, growth delay and seizures [98] (OMIM#194190), isolated *NSD2* deficiency constitutes a distinct disorder [99,100,101]. Conditional deletion of *Nsd2* in the developing neocortex markedly impairs astrocyte production during late embryogenesis and subsequently causes learning and memory deficits [102]. Integrated epigenomic and transcriptomic analyses showed that NSD2 deposits H3K36me2 at the *Egfr* promoter, thereby maintaining *Egfr* expression and downstream ERK signaling during the neurogenic-to-gliogenic transition [102]. Pharmacological restoration of ERK activity rescues astrocyte-production defects both in vitro and in vivo [102]. These findings establish an NSD2–H3K36me2–EGFR–ERK pathway linking chromatin regulation to glial differentiation and demonstrate that NDD-associated SETD proteins can influence cognition through abnormalities in non-neuronal as well as neuronal lineages.

Pathogenic *ASH1L* variants similarly cause a broad syndromic NDD characterized by developmental delay, intellectual disability, substantial speech impairment, and variable autism spectrum disorder, attention deficit hyperactivity disorder, seizures, and sleep disturbances [59] (OMIM#617796) (Table 1). *Ash1l* haploinsufficiency models identify abnormal synaptic remodeling as an important downstream mechanism [103]. *Ash1l* mutant mice exhibit altered social behavior, repetitive behavior, anxiety-related phenotypes, and impaired discrimination associated with excessive synapse formation and defective activity-dependent synapse pruning [103]. In these mice, EphA7 expression is reduced through increased EZH2-dependent H3K27me3 across the *EphA7* locus. Activation of EphA7 signaling with ephrin-A5 improves synapse pruning and rescues discrimination deficits [103], providing direct evidence linking an epigenetically dysregulated target gene to a neuronal and behavioral phenotype. The social and repetitive behavioral abnormalities observed in *Ash1l*-deficient mice correspond broadly to behavioral manifestations reported in individuals with ASH1L-related NDD. Conditional deletion of *Ash1l* in the developing nervous system also produces delayed brain development together with autism spectrum disorder-related abnormalities [104]. RNA-seq during neural progenitor differentiation demonstrated that *Ash1l* loss impairs the normal induction of genes involved in brain development and intercellular communication while also dysregulating genes involved in migration and growth [104]. These findings suggest that not only abnormal synaptic pruning but also failure to establish appropriate developmental transcriptional programs contributes to the behavioral phenotype.

## 4. SUV39/EHMT/SETDB Family

**Figure 5:**
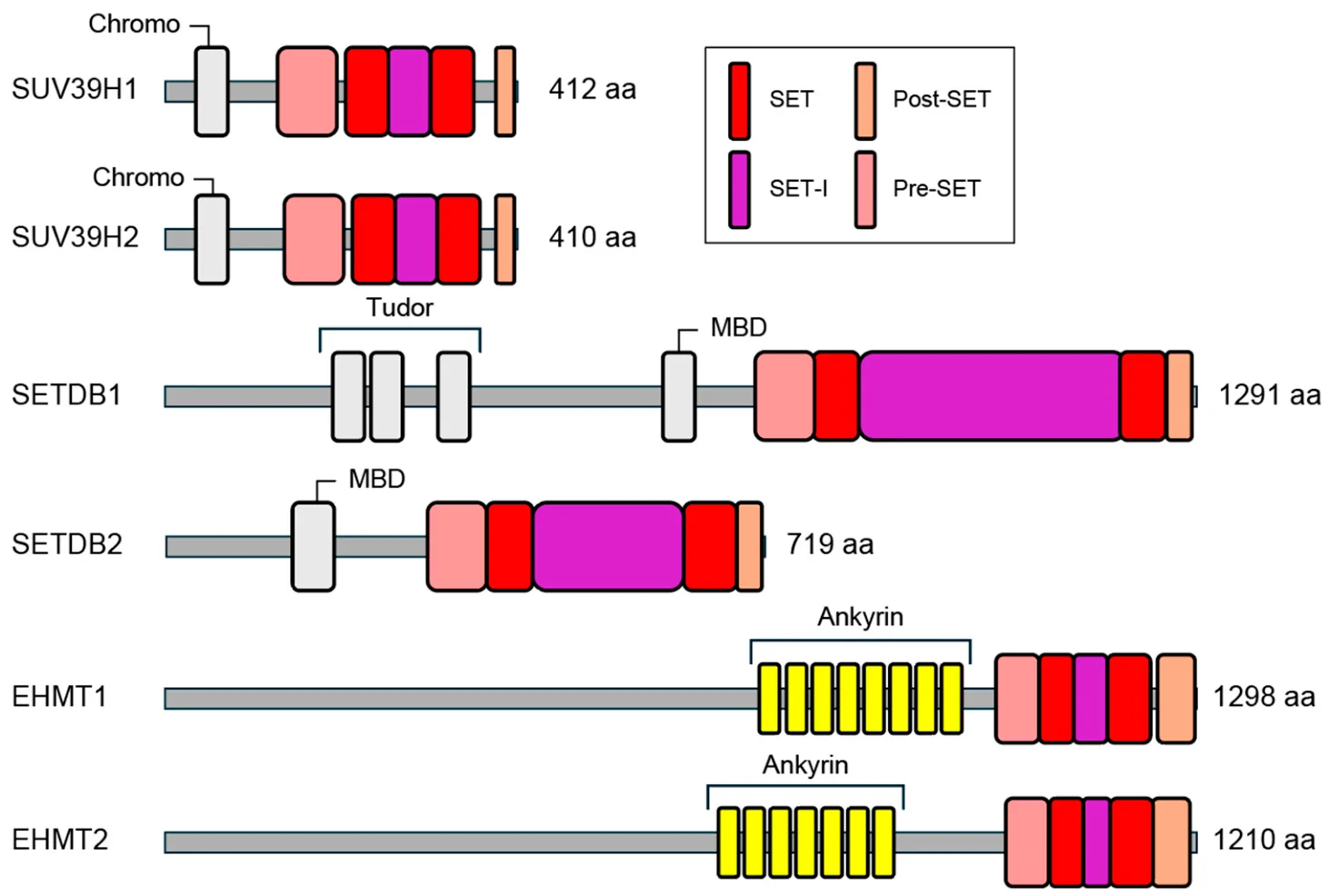
Domain structures of SUV39/EHMT/SETDB family histone methyltransferases. SET domains are indicated by red rectangles, SET-I domains by purple rectangles, post-SET domains by light orange rectangles, pre-SET domains by pink rectangles, and ankyrin repeats by yellow rectangles. Chromodomains (Chromo), Tudor domains, and methyl-CpG-binding domains (MBDs) are indicated by gray rectangles.

Human methyltransferases targeting H3K9, which are evolutionarily related to Drosophila Suppressor of variegation 3–9, include SUV39H1, SUV39H2, EHMT1 (also known as GLP), EHMT2 (also known as G9a), SETDB1, and SETDB2. These proteins constitute distinct evolutionary branches characterized by different accessory-domain architectures [105,106,107] (Figure 5). SUV39 proteins (SUV39H1 and SUV39H2) contain chromodomains, whereas EHMT proteins (EHMT1 and EHMT2) possess ankyrin repeats in addition to their catalytic SET domains. SETDB proteins (SETDB1 and SETDB2) are distinguished by the presence of MBDs. These differences in domain architecture provide mechanisms for directing related H3K9 methyltransferase activities toward distinct chromatin contexts and regulatory pathways. Notably, the ankyrin repeats of EHMT1 and EHMT2 recognize mono- and dimethylated H3K9, enabling these enzymes to couple catalytic activity with recognition of their methylated products and thereby facilitating the establishment and propagation of repressive chromatin states [108].

Among H3K9 methyltransferases, heterozygous loss-of-function variants in *EHMT1* cause Kleefstra syndrome (OMIM# 610253). Its principal features include intellectual disability, markedly impaired expressive speech, childhood hypotonia, and a recognizable facial appearance, with variable autistic behaviors, epilepsy, sleep disturbances, and congenital abnormalities [60,61] (Table 1). Recent evidence has extended this group with de novo heterozygous *EHMT2* variants reported in 2026 in individuals with a Kleefstra-like developmental syndrome and functionally impaired methyltransferase activity [62] (Table 1). Biallelic cases have also been described [109], although the recessive presentation and the relationship between inheritance pattern and molecular mechanism remain less extensively characterized. *Ehmt1* heterozygous mice exhibit learning and memory abnormalities accompanied by reduced dendritic arborization, decreased mature spine density, and altered hippocampal excitatory neurotransmission [110]. Although this model has not been subjected to the same depth of genome-wide neuronal transcriptomic analysis as some H3K4 methyltransferase models, studies of the closely interacting EHMT1/EHMT2 complex have provided important mechanistic insight. Postnatal neuron-specific deletion of *Ehmt1* and *Ehmt2* causes learning, motivational, and adaptive-behavior abnormalities associated with widespread derepression of genes normally characteristic of non-neuronal cells and neuronal progenitors [111]. These findings indicate that H3K9me2-mediated repression is continuously required in mature neurons to prevent inappropriate reactivation of alternative cellular programs. Thus, EHMT1/EHMT2-dependent maintenance of transcriptional identity represents a plausible mechanism linking reduced H3K9 methylation to cognitive dysfunction. The intellectual and learning impairment characteristic of Kleefstra syndrome is broadly paralleled by learning and memory abnormalities in Ehmt1 heterozygous mice. At the mechanistic level, however, these behavioral abnormalities are associated with reduced dendritic arborization, decreased mature spine density, and altered hippocampal excitatory neurotransmission [110], emphasizing that cross-species correspondence is most appropriately interpreted at the level of affected cognitive and neuronal functions rather than as direct equivalence between human intellectual disability and performance in individual mouse behavioral tasks. Together with observations from *Setd1a*- and *Kmt2d*-deficient mice, these findings further demonstrate that apparently similar cognitive outcomes can arise through distinct cellular and molecular pathways.

## 5. EZH Family

**Figure 6:**
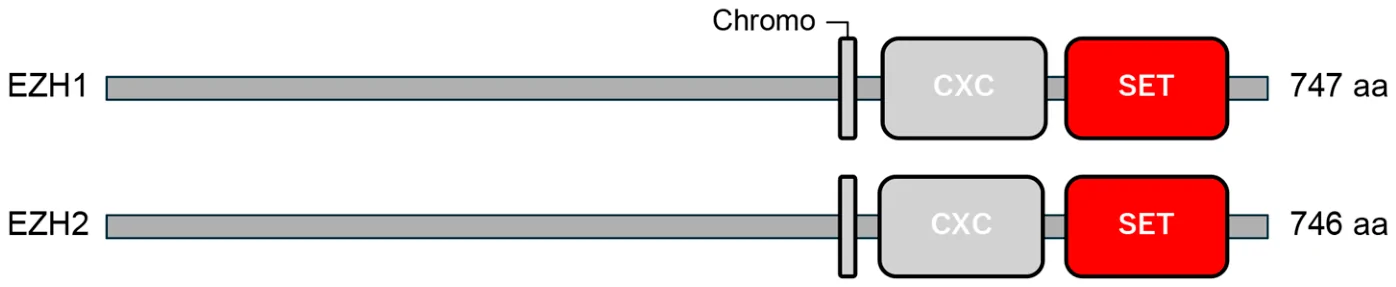
Domain structures of EZH family histone methyltransferases. SET domains are indicated by red rounded rectangles, CXC domains by gray rounded rectangles, and nuclear localization signals (NLSs) by gray rectangles.

The Enhancer-of-zeste family, represented in humans by EZH1 and EZH2, catalyzes H3K27 methylation as the catalytic component of Polycomb repressive complex 2 (PRC2) [112,113] (Figure 6). The architecture of these proteins underscores the functional importance of interactions extending beyond the SET domain itself. Structural analyses of PRC2 have demonstrated that assembly with regulatory partners, including EED and SUZ12, is required to organize and activate the EZH2 catalytic region [114].

In *EZH1*, both heterozygous gain-of-function missense variants and biallelic loss-of-function variants cause NDDs with overlapping developmental and speech impairment [63] (Table 1). In cortical neuron differentiation model of human stem cells, *EZH1* variation alters neuronal differentiation, with EZH1 loss of function enriching progenitor stages and EZH1 gain of function favoring features of differentiated neurons [63]. In contrast, *Ezh1* deletion in mice does not produce overt cellular or molecular brain abnormalities and neurological defects [115]. Thus, despite evidence from human neural models demonstrating an important role for EZH1 in neurogenesis, loss of *Ezh1* alone appears to produce relatively subtle neurological consequences in mice, potentially reflecting species-specific requirements or functional compensation by EZH2. Interpretation of this discrepancy also requires consideration of variant mechanism. Human EZH1-related NDD can result from both heterozygous gain-of-function variants and biallelic loss-of-function variants, whereas conventional *Ezh1* deletion models primarily address the consequences of loss of gene function. To the best of our knowledge, corresponding disease-associated EZH1 gain-of-function variants have not yet been characterized in mouse models.

Heterozygous missense variants in *EZH2* cause Weaver syndrome (OMIM#277590), characterized by overgrowth, advanced bone age, distinctive facial features, camptodactyly, and variable developmental or intellectual impairment [64,65] (Table 1). Functional studies support dominant-negative effects for examined Weaver-associated variants [116]. Loss of EZH2 from early postmitotic neurons delays neuronal migration, increases dendritic complexity and spine density, disrupts synaptic plasticity, and impairs multiple forms of memory [117]. RNA-seq identified extensive transcriptional dysregulation, with affected genes enriched for neuronal migration, neuron projection morphogenesis, differentiation, synapse organization, and dendritic development. Among these targets, *Pak3* and *Bdnf* are upregulated following *Ezh2* deletion [117]. PAK3 was further identified as a direct H3K27me3-regulated target, and inhibition of PAK3 function reverses the increased dendritic spine density caused by *Ezh2* loss. These findings establish a direct mechanistic link between loss of Polycomb-mediated transcriptional repression, inappropriate expression of a neuronal morphogenesis regulator, and altered neuronal structure.

## 6. KMT5 Family

**Figure 7:**
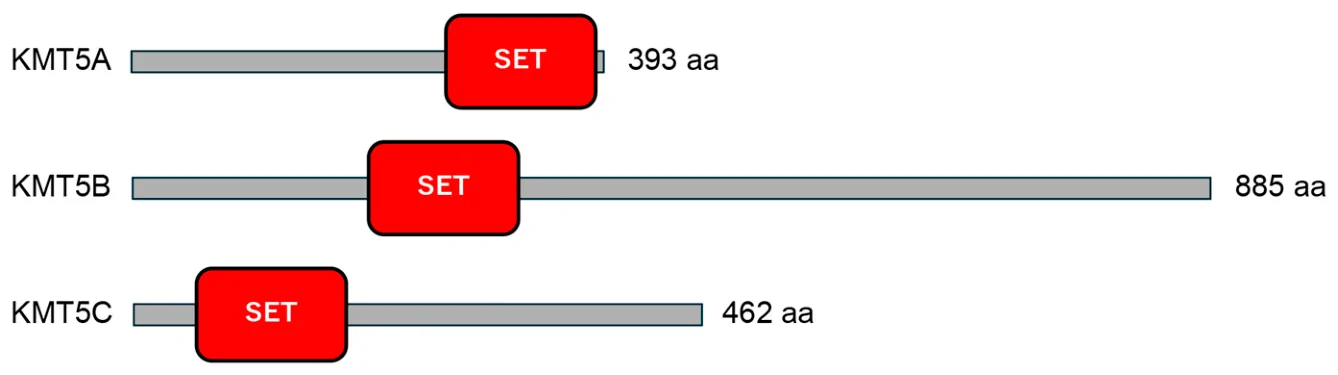
Domain structures of KMT5 family histone methyltransferases. SET domains are indicated by red ellipses.

The H4K20 methyltransferases provide another example of a substrate-based grouping comprising evolutionarily and structurally distinct enzyme families (Figure 7). KMT5A, also known as SETD8 or PR-Set7, primarily catalyzes H4K20 monomethylation, whereas KMT5B (also known as SUV420H1) and KMT5C (also known as SUV420H2) predominantly contribute to the generation of higher H4K20 methylation states [118]. Structural studies of SUV420 enzymes (KMT5B and KMT5C) have revealed specialized interactions with histone H4 and the nucleosome that facilitate substrate recognition and precise positioning of the catalytic machinery [119,120]. Thus, the functional specificity of H4K20 methyltransferases is determined not only by the catalytic properties of their active sites but also by their ability to recognize and engage the surrounding chromatin environment.

Exome sequencing of more than four thousand families containing individuals with undiagnosed developmental disorders found that only one individual carried a de novo synonymous variant in *KMT5C* and no de novo mutations in *KMT5A* [121]. Subsequent case studies of patients with cerebellar tonsillar herniation or autism spectrum disorder reported missense mutations in *KMT5A* [122,123]. However, no constitutional KMT5A- or KMT5C-related disorder had yet been established. In contrast, there is clear evidence that pathogenic heterozygous variants in *KMT5B*, including loss-of-function and missense variants, cause intellectual developmental disorder, autosomal dominant 51, characterized by developmental delay, intellectual disability, autism spectrum disorder, and speech impairment [66,67] (MRD51, OMIM# 617788) (Table 1). Macrocephaly and variable overgrowth are prominent clinical features. Notably, expanded clinical characterization has identified hypotonia, joint hypermobility, congenital heart defects, and other malformations as additional components of the phenotype. Although most pathogenic variants occur de novo, familial transmission has also been documented. These findings highlight the contribution of KMT5B-mediated chromatin regulation to both neurodevelopment and systemic organ development [68] (Table 1). *Kmt5b* haploinsufficient models exhibit developmental growth abnormalities, reduced muscle mass and neuromuscular strength, and defects relevant to the hypotonia and motor delay observed in individuals with KMT5B-associated NDDs [124]. Additional experimental models demonstrate impaired neural progenitor proliferation, accelerated neuronal migration, reduced dendritic complexity, and altered dendritic spine density following KMT5B reduction [125]. Importantly, RNA-seq of *Kmt5b*-haploinsufficient mouse brains identified dysregulated pathways involved in nervous-system development and function, including axon-guidance signaling [68]. These findings suggest that altered developmental transcription contributes alongside peripheral neuromuscular abnormalities to the phenotype associated with *KMT5B* haploinsufficiency. The correspondence between the human and mouse phenotypes is therefore evident across several functional domains: developmental and motor impairment in affected individuals is accompanied by abnormalities in neural progenitor proliferation, neuronal migration, dendritic maturation, and neuromuscular function in experimental models.

## 7. SETD3

SETD3 provides a particularly informative example of a SETD protein that targets a non-histone substrate. Biochemical and structural studies have established that SETD3 catalyzes the methylation of histidine 73 in β- and γ-actins [126,127,128] (Figure 1). This discovery expands the biochemical repertoire of SETD proteins beyond lysine methylation and underscores the importance of experimentally validating substrate specificity rather than inferring enzymatic activity solely from domain architecture. Together with catalytically inactive proteins such as KMT2E, SETD3 illustrates how the conserved SET scaffold has undergone substantial functional diversification, encompassing both altered substrate and amino acid specificity and divergence from canonical histone lysine methyltransferase activity. At present, SETD3 is not an established monogenic neurodevelopmental disorder gene. However, specific pathogenic variants in actin genes are associated with Baraitser–Winter cerebrofrontofacial syndrome (BRWS) (OMIM#243310, #614583), characterized by developmental delay and intellectual disability of variable severity, frequently accompanied by frontal-predominant pachygyria or other cortical malformations, epilepsy, and progressive microcephaly [129,130]. There are two types of BRWS: type 1 (BRWS1) generally exhibits more pronounced craniofacial abnormalities and a broader spectrum of congenital malformations (OMIM#243310), whereas type 2 (BRWS2) tends to present with milder facial dysmorphism but a higher frequency of neuronal migration abnormalities, particularly pachygyria (#614583). Specifically, the *ACTB* gene encoding β-actin is mutated in BRWS1, while the *ACTG1* gene encoding γ-actin is mutated in BRWS2. These mutations affect residues within the SETD3–actin interaction interface, suggesting a potential mechanistic link between SETD3-mediated actin regulation and NDD pathogenesis [131]. However, disease-associated actin variants have not yet been shown to produce a well-established Baraitser–Winter cerebrofrontofacial syndrome phenotype in mouse models, and a direct relationship between SETD3-dependent actin methylation and the neurological manifestations of Baraitser–Winter cerebrofrontofacial syndrome remains to be established. Consequently, unlike several other SETD protein families discussed above, a robust human–mouse genotype–phenotype correspondence cannot yet be defined for SETD3-dependent actin regulation.

## 8. Discussion

Pathogenic variants in SETD genes cause a broad spectrum of clinical manifestations, ranging from relatively mild developmental and behavioral abnormalities to severe syndromic disorders characterized by developmental delay, intellectual disability, speech and language impairment, autism spectrum disorder-related features, epilepsy, growth abnormalities, and congenital anomalies. Although SETD proteins share a conserved catalytic domain, they differ markedly in their histone substrates, domain architecture, interacting partners, genomic targets, and biological functions. The phenotypic overlap among disorders caused by disruption of these functionally diverse proteins therefore highlights the sensitivity of human neurodevelopment to disturbances in epigenetic and transcriptional regulation.

The clinical consequences of SETD gene variants can differ substantially, even among variants affecting the same gene. For example, pathogenic *SETD2* variants have been associated with Luscan–Lumish syndrome (OMIM #616831) and Rabin–Pappas syndrome (OMIM #620155). Although these conditions share neurodevelopmental impairments, they exhibit distinct clinical manifestations, particularly regarding growth abnormalities and congenital anomalies. Luscan–Lumish syndrome is commonly associated with macrocephaly and overgrowth, whereas Rabin–Pappas syndrome is characterized by microcephaly, severe developmental impairment, and multiple congenital anomalies. These differences highlight the importance of considering variant-specific molecular effects when interpreting genotype–phenotype relationships. Similarly, pathogenic variants in *KMT5B* cause a neurodevelopmental disorder characterized by intellectual disability, developmental delay, speech impairment, and variable autism-related features. Expanded clinical characterization has further identified congenital heart defects and other structural malformations as additional components of the KMT5B-associated phenotype, underscoring its broader clinical heterogeneity.

Studies using genetically modified mice have substantially advanced our understanding of the roles of SETD proteins in nervous system development. Across multiple models, abnormalities in neural progenitor function, neuronal differentiation and maturation, learning and memory, social behavior, synaptic function, and neuronal excitability have been reported. Comparison with human phenotypes reveals substantial conservation in several neurodevelopmental domains, supporting the utility of mouse models for mechanistic studies. However, as illustrated by the absence of generalized overgrowth in *Nsd1* heterozygous mice [96] and the limited recapitulation of progressive dystonia in forebrain-specific *Kmt2b* models [70], some genetically relevant models may reproduce only selected functional dimensions of the corresponding human disorder. Mouse models should therefore be viewed not as complete replicas of human disorders but as experimental systems for identifying conserved biological consequences of SETD protein dysfunction.

Although deletion of genes is easily linked to loss of function, missense variants require careful functional characterization because they may result in loss-of-function, gain-of-function, dominant-negative, or more selective domain- or substrate-specific effects. The examples of SETD2, EZH1, and EZH2 discussed above illustrate this principle [63,94,95,116,117]: disease-associated variants within the same gene can produce molecular consequences that differ substantially from those generated by simple gene deletion. Moreover, the SETD2 SRI-domain mutant demonstrates that neurobehavioral abnormalities can arise through impaired methylation of a non-histone substrate, α-tubulin, even in the absence of prominent alterations in H3K36 methylation or gene expression [95]. Integrating human genetic data with biochemical, epigenomic, transcriptomic, proteomic, and cellular analyses will therefore be essential for establishing robust genotype–molecular phenotype–clinical phenotype relationships.

An important challenge for future studies is whether genetically distinct SETD-related NDDs converge on common molecular pathways. The overlapping clinical phenotypes observed across these disorders are unlikely to result from a uniform alteration in histone methylation because individual SETD proteins target different histone residues and can exert opposing effects on transcription. Instead, distinct epigenetic abnormalities may converge on neurodevelopmentally critical gene networks and cellular processes. The mechanistic studies summarized above support this concept while also emphasizing substantial gene-specificity. For example, NSD1-dependent H3K36me2 contributes to maintenance of cortical neuronal identity [97], NSD2 regulates gliogenic differentiation through an H3K36me2–EGFR–ERK pathway [102], and ASH1L influences activity-dependent synaptic remodeling in part through regulation of EphA7 [103,104]. *SETD5* deficiency, by contrast, affects not only transcriptional regulation but also ribosome biogenesis and global protein synthesis [81,82,86,87]. Thus, convergence among SETD-related NDDs may occur at the level of cellular functions even when the upstream molecular pathways are distinct. Comparative analyses across different SETD genes at the transcriptomic, epigenomic, translatomic, and proteomic levels will be important for identifying such shared pathways and distinguishing them from gene-specific mechanisms.

**Figure 8:**
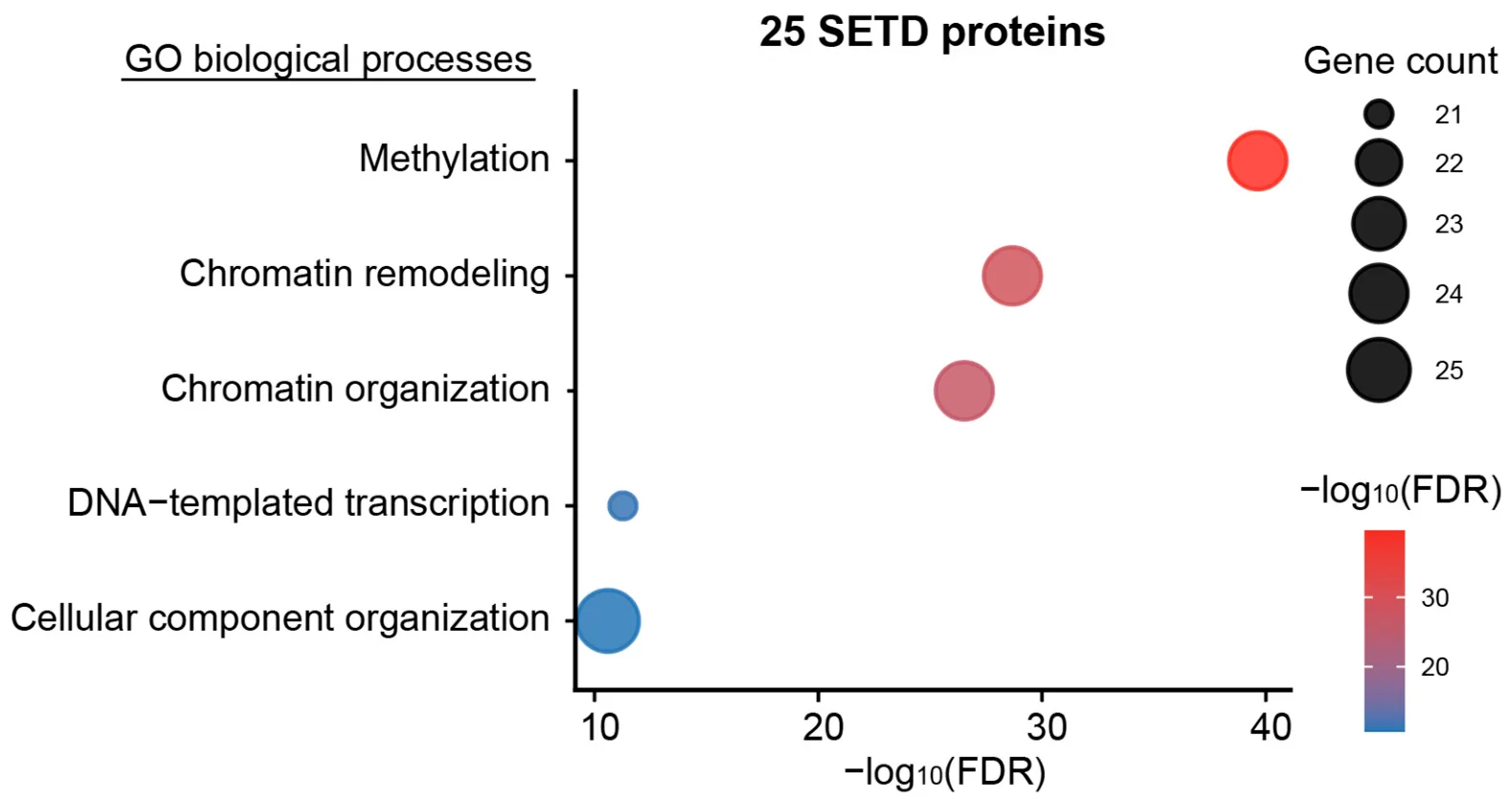
Gene Ontology enrichment analysis of 25 SETD proteins discussed in this review. Bubble plot showing the five most significantly enriched Gene Ontology (GO) biological process terms associated with the analyzed SETD proteins. The horizontal axis represents the negative logarithm of the false discovery rate (−log10 FDR), while bubble size indicates the number of genes associated with each GO term. Bubble color represents enrichment significance, with red indicating greater statistical significance. GO terms are arranged in descending order of statistical significance.

To systematically investigate the functional relationships among SETD proteins discussed in this review, we performed Gene Ontology (GO) enrichment analysis using the GO Biological Process database (Figure 8). The five highest-ranking enriched terms were methylation (GO:0032259), chromatin remodeling (GO:0006338), chromatin organization (GO:0006325), DNA-templated transcription (GO:0006351), and cellular component organization (GO:0016043). These findings are consistent with the established roles of SETD proteins in epigenetic regulation, chromatin organization, and transcriptional control. Notably, the enrichment of these functionally related biological processes suggests that SETD proteins may contribute to common regulatory networks despite their differences in substrate specificity, catalytic activity, and molecular functions. However, because these proteins were selected based on their SET domains and relevance to NDDs, the observed enrichment should be interpreted cautiously and does not necessarily indicate convergence on identical downstream molecular pathways. Nevertheless, these findings provide a functional framework for understanding how disruption of distinct SETD proteins may contribute to overlapping neurodevelopmental phenotypes.

An additional perspective on cross-gene convergence emerges from the contrasting growth phenotypes associated with SETD-related disorders. Pathogenic variants in *NSD1*, *SETD2*, *EZH2*, and *KMT2E* are associated with overgrowth and/or macrocephaly [49,50,56,64,65], whereas *NSD2* deficiency is associated with growth restriction [57,58] and the SETD2 p.Arg1740Trp variant causes a distinct disorder characterized by microcephaly [55]. These observations suggest that SETD proteins participate in regulatory pathways controlling developmental growth, although disruption of individual components can produce opposing phenotypic consequences. One potential molecular framework involves the interplay between H3K36 methylation, DNA methylation, and PRC2-mediated H3K27 methylation. H3K36me2 and H3K36me3 contribute to the recruitment and genomic distribution of DNA methyltransferases DNMT3A and DNMT3B, respectively [132,133,134,135], while H3K36 methylation antagonizes PRC2-mediated H3K27 methylation [13,136]. Consequently, alterations in H3K36 methylation may influence both DNA methylation patterns and Polycomb-dependent transcriptional repression, potentially affecting developmental gene-expression programs that regulate cellular proliferation and differentiation. However, the precise contribution of these interactions to the opposing growth phenotypes observed in individual SETD-related disorders remains to be established.

The functional relationship between H3K36 and H3K27 methylation is also relevant to neuronal development. As discussed above, *ASH1L* deficiency increases EZH2-dependent H3K27me3 at the *EphA7* locus, resulting in reduced EphA7 expression and impaired activity-dependent synaptic pruning [103]. This finding provides an example of how disruption of an H3K36 methyltransferase-associated regulatory pathway can alter PRC2-mediated transcriptional repression and downstream neuronal function. Together with the developmental abnormalities associated with NSD1, NSD2, SETD2, and EZH2 dysfunction, these observations suggest that interactions among histone methylation pathways may represent an important level of molecular convergence.

DNA methylation episignatures provide another complementary framework for investigating convergence and divergence among SETD-related NDDs [137]. Genome-wide DNA methylation profiling has identified characteristic episignatures in several disorders caused by pathogenic variants in chromatin-regulatory genes, including SETD-related disorders such as those associated with *KMT2C*, *NSD1*, and *EZH2* [45,138,139]. These profiles reflect reproducible alterations in DNA methylation patterns and may provide molecular biomarkers for variant interpretation and disease classification [140]. Integrating DNA methylation profiling with histone modification maps, transcriptomic analyses, and functional studies in patient-derived neural models will therefore be important for establishing the biological significance of these molecular relationships.

The developmental timing and cellular context of SETD dysfunction also remain important areas for investigation. Conditional and inducible animal models will help define the neural cell populations and developmental periods in which individual SETD proteins are required. Studies discussed above indicate that SETD proteins can act at markedly different developmental and cellular stages, ranging from regulation of neural progenitor differentiation and cortical arealization to maintenance of transcriptional identity and synaptic function in mature neurons. The contribution of non-neuronal cell types should also receive greater attention, as illustrated by the requirement for NSD2 in astrocyte production [102] and the reported effects of *KMT2E* haploinsufficiency on microglial phagocytosis and synaptic clearance [79]. Such findings suggest that SETD-related NDDs may arise not only from neuron-intrinsic abnormalities but also from disruption of interactions among multiple neural cell types.

Of particular therapeutic relevance is whether neurological phenotypes caused by SETD protein dysfunction become irreversibly established during embryonic development or remain dependent on ongoing molecular abnormalities in the postnatal or adult brain. The studies summarized here provide several encouraging examples of functional reversibility. Restoration of *Setd1a* expression in adulthood improves working-memory deficits [39], whereas developmental restoration of *Kmt2a* ameliorates neurological phenotypes [72]. Pharmacological inhibition of LSD1 improves behavioral, morphological, or transcriptional abnormalities in *Setd1a*-, *Kmt2c*-, and *Kmt2d*-deficient mice [39,75,77], while histone deacetylase inhibition also ameliorates chromatin and neurogenic abnormalities in *Kmt2d* mutant mice [76]. However, the molecular mechanisms underlying the beneficial effects of LSD1 inhibition may differ among these models. LSD1 primarily demethylates H3K4me1 and H3K4me2 but cannot directly remove H3K4me3 [141,142]. Therefore, although SETD1A predominantly catalyzes H3K4 trimethylation [30,31,32], the beneficial effects of LSD1 inhibition in *Setd1a*-deficient mice are unlikely to result from direct restoration of H3K4me3 through inhibition of its demethylation. Instead, these effects may involve modulation of H3K4me1/2 at shared regulatory elements, including enhancers, and subsequent normalization of dysregulated transcriptional programs. In contrast, KMT2C and KMT2D primarily regulate enhancer-associated H3K4 monomethylation [32,33,34], suggesting that LSD1 inhibition may more directly counteract their deficiency by preserving H3K4me1 at affected regulatory regions. Nevertheless, the precise molecular mechanisms responsible for the observed phenotypic improvements remain to be fully elucidated. Furthermore, LSD1 also regulates non-histone substrates [143], and its neuron-specific isoform, LSD1n, exhibits distinct substrate specificities and contributes to neuronal differentiation and activity-dependent gene expression [144,145,146]. These properties indicate that pharmacological LSD1 inhibition may affect multiple chromatin-associated and non-histone regulatory pathways, potentially influencing neuronal functions independently of H3K4 methylation.

Importantly, therapeutic opportunities may not be restricted to correction of transcriptional abnormalities. As described above, SETD5 insufficiency reduces rDNA transcription, ribosome biogenesis, and global protein synthesis, indicating that specific abnormalities in translational capacity may represent downstream targets independently of, or in addition to, transcriptional dysregulation. Likewise, restoration of ERK signaling rescues impaired astrocyte production following *Nsd2* loss, and activation of EphA7 signaling ameliorates abnormal synaptic pruning associated with *Ash1l* deficiency. These findings suggest that targeting well-defined downstream molecular or cellular abnormalities may sometimes be more feasible than directly correcting the primary chromatin-regulatory defect. More broadly, the identification of abnormalities in translation, cytoskeletal regulation, synaptic remodeling, glial differentiation, or cellular signaling emphasizes that SETD-related NDDs should not be considered exclusively as disorders of transcription. Determining which downstream alterations are causal, reversible, and sufficiently selective for therapeutic intervention will therefore be an important goal of future studies.

Future studies should also increasingly incorporate human experimental systems. Patient-derived induced pluripotent stem cells, differentiated neural cells, and brain organoids provide opportunities to investigate human-specific aspects of SETD dysfunction that cannot be adequately reproduced in mice. This is particularly important for genes such as EZH1, for which human neural models reveal substantial effects of pathogenic variants on neuronal differentiation despite relatively subtle phenotypes following *Ezh1* loss in mice. Genome editing can further enable direct comparison of patient-derived variants in otherwise isogenic genetic backgrounds and allow loss-of-function, gain-of-function, dominant-negative, and substrate-specific mechanisms to be distinguished experimentally. Integration of these approaches with animal models will provide a broader insight into how disruption of epigenetic regulation propagates through transcriptional, translational, and cellular networks to produce abnormal neurodevelopment as well as a powerful framework for distinguishing evolutionarily conserved mechanisms from human-specific consequences of SETD gene dysfunction.

## Figures and Tables

**Table 1:** Neurodevelopmental disorders associated with pathogenic variants in human SETD genes. OMIM, online mendelian inheritance in man; AD, autosomal dominant; AR, autosomal recessive; ASD, autism spectrum disorder; DD, developmental delay; ADHD, attention deficit hyperactivity disorder; GoF, gain of function; ID, intellectual disability; LoF, loss of function; NDD, neurodevelopmental disorder. Inheritance/variant type, major phenotypes, and other characteristic features are derived from the OMIM phenotype number which is shown in the column designated as OMIM number.

Gene	Syndrome/Disorder	OMIM Number/Relevant Reference	Inheritance/Variant Types	Major Phenotypes	Other Characteristic Features
*SETD1A*	SETD1A-related NDD	619056 [38]	AD; predominantly de novo; LoF	DD/ID, prominent speech and language impairment, behavioral abnormalities, variable epilepsy	Variable psychiatric manifestations
*SETD1B*	SETD1B-related NDD	619000 [41]	AD; predominantly de novo; LoF	DD/ID, severe language impairment, ASD/behavioral abnormalities, epilepsy	Developmental impairment may precede epilepsy
*KMT2A*	Wiedemann–Steiner syndrome	605130 [42,43]	AD; predominantly de novo; LoF	DD/ID, speech impairment, hypotonia	Hypertrichosis cubiti, short stature, feeding difficulties, characteristic facial features
*KMT2B*	KMT2B-related dystonia/NDD	617284, 619934 [44]	AD; predominantly de novo; LoF	DD/ID, variable speech impairment, microcephaly; childhood-onset dystonia is frequent	Progressive generalized dystonia, growth restriction
*KMT2C*	KMT2C-related NDD	617768 [45]	AD; predominantly LoF, predominantly de novo	DD/ID, language impairment, ASD and behavioral/psychiatric abnormalities, variable seizures	Variable dysmorphic and congenital features
*KMT2D*	Kabuki syndrome type 1	147920 [46,47,48]	AD; predominantly LoF, predominantly de novo	DD, mild-to-moderate ID, speech/language difficulties	Characteristic facial features, persistent fingertip pads, skeletal abnormalities, growth deficiency, cardiac/auditory/immune abnormalities
*KMT2E*	O’Donnell–Luria–Rodan syndrome	618512 [49,50]	AD; predominantly LoF, predominantly de novo	DD/ID, speech delay, hypotonia, variable ASD and epilepsy	Macrocephaly; some missense variants associated with more severe phenotypes including microcephaly and epileptic encephalopathy
*SETD5*	SETD5-related NDD	615761 [51,52]	AD; predominantly LoF, predominantly de novo	ID, speech and motor delay, hypotonia, behavioral abnormalities, variable autistic features	Dysmorphism, feeding difficulties, congenital anomalies
*SETD2*	Luscan–Lumish syndrome	616831 [53,54]	AD; predominantly LoF, predominantly de novo	DD/ID, variable ASD	Macrocephaly and variable overgrowth
*SETD2*	Rabin–Pappas syndrome	620115 [55]	AD; specific missense variants, particularly p.Arg1740Trp; predominantly de novo	Severe-to-profound DD/ID, seizures	Microcephaly, brain malformations, multiple congenital anomalies
*SETD2*	Intellectual developmental disorder, autosomal dominant 70 (MRD70)	620157 [55]	AD; reported de novo; specific missense variants, particularly p.Arg1740Gln; predominantly de novo	Moderate-to-severe intellectual disability, speech difficulties, behavioral abnormalities, occasionally hypotonia	Generally normal growth, low-normal head circumference, absence of major congenital anomalies
*NSD1*	Sotos syndrome	117550 [56]	AD; predominantly LoF, predominantly de novo	Learning difficulties/ID, variable developmental delay	Overgrowth, macrocephaly, advanced bone age, characteristic facial features
*NSD2*	Rauch–Steindl syndrome	619695 [57,58]	AD; predominantly LoF, predominantly de novo	DD, speech and motor delay, variable ID	Prenatal/postnatal growth restriction
*ASH1L*	ASH1L-related NDD	617796 [59]	AD; predominantly LoF, predominantly de novo	DD/ID, marked speech impairment, ASD, ADHD, variable seizures	Sleep disturbances and variable dysmorphic features
*EHMT1*	Kleefstra syndrome	610253 [60,61]	AD; predominantly LoF, predominantly de novo	ID, severe expressive speech impairment, hypotonia, ASD/behavioral abnormalities, variable epilepsy	Characteristic facial features, sleep disturbances, congenital abnormalities
*EHMT2*	EHMT2-related NDD/Kleefstra-like syndrome	not yet assigned [62]	AD; predominantly LoF, predominantly de novo; biallelic variants also reported	DD/ID, speech impairment and variable behavioral abnormalities	Phenotypic spectrum remains incompletely defined
*EZH1*	EZH1-related NDD	not yet assigned [63]	AD GoF or AR LoF; predominantly de novo	DD/ID, prominent speech impairment	Phenotypic severity and additional features vary according to molecular mechanism
*EZH2*	Weaver syndrome	277590 [64,65]	AD; predominantly missense variants; predominantly de novo	Variable DD/ID	Prenatal/postnatal overgrowth, advanced bone age, characteristic facial features, camptodactyly
*KMT5B*	Intellectual developmental disorder, autosomal dominant 51 (MRD51)	617788 [66,67,68]	AD; predominantly LoF, occasionally likely pathogenic missense variant (p.His181Asp, p.Asn278Ile), predominantly de novo, occasionally inherited	DD/ID, speech and motor impairment, hypotonia, variable ASD and seizures	Frequent macrocephaly, congenital heart defects and other malformations

## Data Availability

No new data were created or analyzed in this study. Data sharing is not applicable to this article.
